# The Role of Preoperative Virtual Reality for Anxiety Reduction in Pediatric Surgical Patients: A Systematic Review and Meta-Analysis

**DOI:** 10.7759/cureus.77077

**Published:** 2025-01-07

**Authors:** Anas E Ahmed, Lojain I Daak, Mazen A Alayidh, Rahaf R Filfilan, Raneem M Alathath, Anmar A Rehbini, Thekra A Alshabrami, Saad A Alqahatani, Renad A Alzahrani, Hawazin S Althobaiti

**Affiliations:** 1 Community Medicine, Jazan University, Jazan, SAU; 2 College of Medicine, Jazan University, Jazan, SAU; 3 College of Medicine, King Khalid University, Abha, SAU; 4 College of Medicine, Ibn Sina National College For Medical Studies, Jeddah, SAU; 5 College of Medicine, Umm Al-Qura University, Makkah, SAU

**Keywords:** anxiety management, emergence delirium, non-pharmacological interventions, pediatric surgical patients, preoperative anxiety, randomized controlled trials, virtual reality

## Abstract

Preoperative anxiety is a common and significant issue in pediatric surgical patients, often leading to adverse postoperative outcomes such as increased pain, delayed recovery, and emergence delirium. Traditional methods to address this anxiety, including pharmacological interventions like midazolam, can cause undesirable side effects while non-pharmacological approaches, such as parental presence and cognitive-behavioral therapies, require substantial resources. Virtual reality (VR) has emerged as a promising, non-invasive intervention that immerses children in engaging and distracting virtual environments to reduce anxiety. This systematic review and meta-analysis evaluated the effectiveness of VR in alleviating preoperative anxiety in pediatric patients compared to other interventions. A systematic search of randomized controlled trials (RCTs) was conducted using databases such as PubMed, Scopus, Cochrane Library, and Web of Science. Studies involving pediatric patients aged 4-12 years undergoing surgery and using VR were included. The primary outcome was a reduction in preoperative anxiety levels measured by validated scales such as the Modified Yale Preoperative Anxiety Scale (m-YPAS). Secondary outcomes included emergence delirium, postoperative behavioral disturbances, and parental anxiety. Five RCTs involving 356 pediatric patients met the inclusion criteria, with three studies contributing to the meta-analysis. Results demonstrated that VR interventions significantly reduced preoperative anxiety in children compared to control groups, with a pooled standardized mean difference of -0.73 (95% CI: -1.15 to -0.31, p < 0.001). VR was particularly effective when used shortly before surgery and in children aged 5-10 years. No significant differences were observed in the incidence of emergence delirium, postoperative behavioral disturbances, or parental anxiety between VR and control groups. These findings indicate that VR is an effective, safe, and non-invasive tool for managing preoperative anxiety in pediatric surgical patients. Further research is needed to assess the long-term effects of VR and standardize its implementation across different surgical settings. Integrating VR into routine preoperative care could significantly improve the surgical experience for children and their families.

## Introduction and background

Preoperative anxiety is a common and significant issue in pediatric patients undergoing surgery. Research shows that up to 75% of children experience moderate to severe anxiety before surgery, which can manifest as behavioral distress, physiological changes, and emotional disturbances [1]. This anxiety can lead to adverse postoperative outcomes, including increased pain perception, prolonged recovery times, and negative behavioral effects [2]. The occurrence of preoperative anxiety is not restricted to any one type of surgery, as children undergoing both major procedures, such as cardiac surgery, and minor procedures, such as dental extractions, are susceptible to stress [3].

Various types of surgeries, including tonsillectomies, hernia repairs, and dental treatments, have been associated with heightened levels of preoperative anxiety in children [4]. Even minimally invasive procedures, such as laparoscopic surgeries and minor day surgeries, can provoke significant anxiety, especially in younger children [5]. The prevalence of anxiety across different surgical types underscores the importance of effective interventions to manage this psychological burden.

Several approaches have been implemented to address preoperative anxiety in pediatric patients, ranging from pharmacological to non-pharmacological strategies [2]. Pharmacological methods, such as the use of sedatives like midazolam, are effective but come with risks, including respiratory depression, paradoxical reactions, and delayed recovery [6]. Non-pharmacological interventions, including parental presence, play therapy, and cognitive-behavioral techniques, are safer alternatives but often require significant time and resources, limiting their applicability in busy surgical settings [3].

Virtual reality (VR) has emerged as a promising non-invasive tool for reducing preoperative anxiety in pediatric patients. VR can transport children to immersive environments, effectively distracting them from the hospital setting and reducing their perceived stress [7]. Unlike pharmacological interventions, VR has no known side effects and can be easily integrated into preoperative protocols, making it an appealing option for healthcare providers [5].

The effectiveness of VR in reducing anxiety has been demonstrated in various medical settings. For instance, VR has been used successfully to reduce anxiety in children undergoing dental procedures, where it significantly lowered distress levels compared to traditional methods [4]. Similarly, VR has been shown to decrease anxiety in children undergoing minor surgical procedures, such as venipuncture and intravenous cannulation, highlighting its broader applicability [8,9].

Not only does VR benefit children, but it can also alleviate parental anxiety. Parents often experience significant stress when their children are scheduled for surgery, and their anxiety can influence the child’s own stress levels [2]. By using VR to reduce the child’s anxiety, parental stress can also be diminished, leading to a more relaxed preoperative environment for both children and their families [7]. Additionally, VR could be used to educate parents about surgical procedures, further reducing their anxiety and improving overall satisfaction with the experience [10].

Despite the promising evidence, VR remains underutilized in pediatric preoperative care, and more research is needed to evaluate its effectiveness across different surgical settings. While the current studies show encouraging results, most focus on specific procedures or involve small sample sizes, limiting the generalizability of the findings. Therefore, a systematic review and meta-analysis are necessary to provide a comprehensive overview of the effectiveness of VR in reducing preoperative anxiety in pediatric patients, which this paper aims to address.

## Review

Methodology

Literature Search Strategy

We followed the Preferred Reporting Items for Systematic Reviews and Meta-Analyses (PRISMA) guidelines during the conduct and reporting of this review [11]. Our search strategy encompassed four major online databases: PubMed, Web of Science (WOS), Scopus, and the Cochrane Central Register of Controlled Trials (CENTRAL), covering the period from inception until November 5, 2024. We employed specific keywords, including "virtual reality", "game-based", "preoperative", "perioperative", and "presurgery". These keywords were combined using Boolean operators, and the search strategy was tailored to each database accordingly. Filters were applied to include only English articles involving human participants. Additionally, we manually scrutinized the reference lists of the included studies to identify any relevant articles missed during the initial search process.

Eligibility Criteria

The eligibility criteria were based on the PIOCS framework (P-population, I-intervention, C-comparison, O-outcome, S-study design). Only English randomized clinical trials were included if they involved children undergoing different types of surgeries, used VR either alone or as adjuvant therapy, compared VR to placebo, no intervention, or any other type of analgesic, and measured any outcome to assess the effect of the intervention. Studies were excluded if they were observational in design, published in languages other than English, or presented only as abstracts without full-text articles.

Study Selection

Two reviewers independently screened the titles and abstracts of the retrieved articles using predetermined eligibility criteria. Any disagreements or discrepancies were resolved by a third reviewer until a consensus was reached.

Data Extraction

The full text of the included articles was further analyzed, and the following data were extracted: sample size, VR technology used, type of surgeries conducted for the children, outcome measures, and main results. Any potential conflicts were resolved by a third reviewer.

Quality Appraisal

The methodological quality of the included studies was independently assessed by two reviewers using the modified Downs and Black scale for clinical trials [12]. This scale consists of 27 questions rating four categories: reporting, external validity, internal validity, and power. Studies were categorized as being of excellent quality if their final scores ranged from 26 to 28, good quality if scores ranged from 20 to 25, fair quality if scores ranged from 15 to 19, and poor quality if scores were 14 or less. Any disagreements or discrepancies were resolved through discussion until a consensus was reached.

Data Synthesis and Analysis

Meta-analysis was conducted if there were at least two studies comparing the efficacy of VR on the same outcome. Standardized mean difference (SMD), 95% confidence interval (CI), and p-value were calculated by comparing the changes in outcomes between the VR and control groups using the random-effects model of analysis. Heterogeneity in treatment effects was examined by calculating the I² index. The level of significance was set at a p-value of up to 0.05. All meta-analyses were carried out using the Comprehensive Meta-Analysis version 2.2.064 software package (Biostat, Englewood, New Jersey, USA).

Results

Study Selection

**Figure 1:**
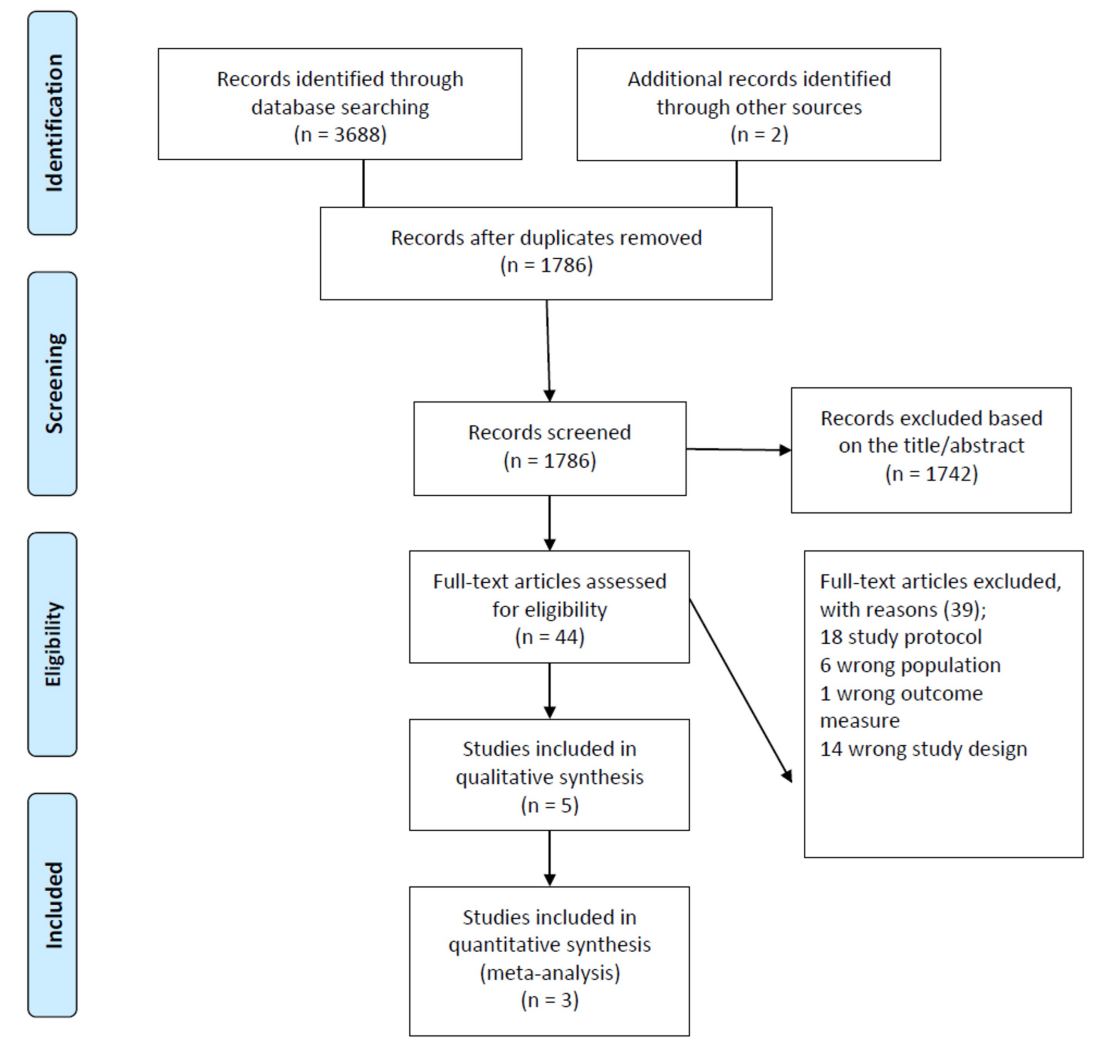
PRISMA flowchart of studies search and selection PRISMA: Preferred Reporting Items for Systematic Reviews and Meta-Analyses

A total of 3,688 records were initially identified through database searches, along with 2 additional records from other sources, yielding a total of 3,690 records. After duplicates were removed, 1,786 records remained for screening. During the title and abstract screening, 1,742 studies were excluded based on relevance, leaving 44 full-text articles for eligibility assessment. Of these, 39 articles were excluded for reasons such as being study protocols, focusing on populations outside the target demographic, employing outcome measures misaligned with the review objectives, or having incompatible study designs. Following this process, five studies met the inclusion criteria for qualitative synthesis, with three of these further included in the quantitative synthesis (meta-analysis) due to meeting the statistical comparison requirements (Figure 1).

Study Characteristics

The included studies were randomized controlled trials (RCTs) conducted in diverse settings across countries like South Korea, Iran, India, and the USA (Table 1). These studies implemented VR interventions aimed at reducing preoperative anxiety or improving procedural outcomes in pediatric surgical patients. The types of VR interventions varied, with examples including immersive VR tours of the operating room using popular characters like Pororo [5,13], VR environments simulating the surgical journey [14], interactive games [15], and calming activity videos such as swimming with dolphins [16].

**Table 1:** Baseline characteristics of the included studies RCT: Randomized Controlled Trial, VR: Virtual Reality, E1: Experimental Group 1, E2: Experimental Group 2, CG: Control Group, GA: General Anesthesia, IQR: Interquartile Range, m-YPAS: Modified Yale Preoperative Anxiety Scale, ED: Emergence Delirium, BAI: Beck Anxiety Inventory, EMLA: Eutectic Mixture of Local Anesthetics, IV: Intravenous

First Author, Year	Country	Design	Type of Surgery	Sample Size	Age (Years)	Type of VR
Jung-Hee Ryu, 2017 [5]	South Korea	RCT	Otolaryngeal (ventilation tube insertion, tonsillectomy, adenoidectomy), ophthalmic, orthopedic, and dental procedures	80 (E1; 41 CG; 39)	4–10 (Median: 6)	Immersive virtual reality (4-minute 360° VR tour of the operating theater using the Pororo character)
Fateme Dehghan, 2019 [14]	Iran	RCT	Abdominal surgery	40 (E1; 20 CG; 20)	7 ± 2	Virtual reality by applying eyeglasses, which provide a wide field of vision and proper vision for the patient. The patient was placed in front of a computer monitor to present the simulated steps of going to the operating room. A headphone was placed on the patient’s ears, and the simulated sounds of entering the virtual environment were played through the headphones.
Jung-Hee Ryu, 2022 [13]	South Korea	RCT	Benign soft mass excision, inguinal hernia repair, central catheter insertion, frenotomy	105 (E1; 35, E2; 35, CG; 35)	E1: 5.0 (IQR 5.0; 6.0) years, E2: 6.0 (IQR 4.0; 7.5) years, and E3: 5.0 (IQR 5.0; 6.5)	VR tour of an operating room (Pororo animation) shown either a few days before surgery or 10 minutes before
Rishabh Ravi, 2023 [16]	India	RCT	Elective surgeries	60 (E1; 30 CG; 30)	8.242 ± 1.2048	Immersive VR (videos such as swimming with dolphins or playing cricket)
Michael J. Jung, 2021 [15]	USA	RCT	Elective surgeries requiring general anesthesia (GA) in pediatric patients	71 (E1: 34, CG; 37)	8.0 ± 2.3 years	Samsung Gear VR headset displaying a pre-selected, interactive game designed for pediatric perioperative use (Mighty Immersion, Palo Alto, CA)

Sample sizes ranged from 40 to 105 participants, with age groups typically spanning 4 to 10 years. For instance, median ages in Jung-Hee Ryu’s studies were 6 years (2017) [5] and 5.0-7.5 years (2022) [13] while average ages in studies by Rishabh Ravi (2023) [16] and Michael J. Jung (2021) [15] were 8.24 and 8.0 years, respectively. Primary outcomes assessed across studies included preoperative anxiety, emergence delirium (ED), postoperative behavioral disturbances, and pain management. Tools like the modified Yale Preoperative Anxiety Scale (m-YPAS) were frequently used. Across the studies, VR interventions consistently showed effectiveness in reducing preoperative anxiety. For example, VR significantly reduced m-YPAS scores in Jung-Hee Ryu’s 2017 [5] and 2022 [13] studies and Michael J. Jung’s 2021 [15] studies. However, impacts on other outcomes, such as postoperative behavioral disturbances and caregiver anxiety, were less consistent, though caregiver satisfaction was generally higher in VR groups.

Quality Assessment

Quality appraisal revealed a range of strengths and weaknesses across the studies. Most studies excelled in reporting outcomes and patient characteristics, although reporting of adverse events was often lacking. External validity scores were lower, indicating limited generalizability due to sample and setting constraints. Internal validity related to bias was generally satisfactory, although blinding participants was challenging due to the nature of the intervention. Randomization and baseline comparability were strengths, but confounding adjustments were inconsistent. Statistical power was sufficient in most studies, except for one that lacked adequate power. Overall, quality scores varied, with some studies achieving strong ratings in methodological rigor while others showed gaps in external validity and reporting adverse events (Table 2).

**Table 2:** Methodological quality assessment of included studies using the modified Downs and Black scale This table presents the methodological quality assessment of included studies using the modified Downs and Black scale. The scale evaluates four domains: reporting (clarity and completeness of study details), external validity (generalizability of results), internal validity - bias (minimization of potential biases in study design and conduct), and internal validity - confounding (adjustment for confounding variables and appropriate randomization). Power assesses whether the study had an adequate sample size to detect significant effects. The total score is the sum across all domains, with a maximum possible score of 28. Studies are categorized as excellent quality (26–28), good quality (20–25), fair quality (15–19), or poor quality (≤14) based on their total score.

Study	Reporting	External Validity	Internal Validity - Bias	Internal Validity - Confounding	Power	Total Score	Quality Category
Contreras, 2018 [10]	8	3	6	3	1	21	Good
Roberts, 2019 [9]	9	3	5	3	0	19	Fair
Kario, 2021 [15]	10	2	5	6	1	24	Good
Baron, 2019 [14]	8	0	4	4	0	16	Fair
Pletcher, 2022 [16]	8	1	5	4	1	19	Fair
Morawski, 2018 [7]	8	3	5	5	1	22	Good

Effect of Interventions

In qualitative synthesis, VR interventions demonstrated notable reductions in preoperative anxiety (Table 3). For example, in the 2017 study by Jung-Hee Ryu [5], significant reductions in m-YPAS scores were observed in the VR group compared to controls, with median scores showing a marked difference. The 2022 study by the same author [13] further highlighted differences among groups receiving VR interventions at various times, with those exposed earlier showing greater anxiety reductions. Similarly, Michael J. Jung’s 2021 study reported significantly lower m-YPAS scores in the VR group at the time of anesthesia induction compared to controls [15]. Other outcomes, like postoperative behavioral disturbances, were less consistently impacted, with some studies reporting no significant differences between VR and control groups. Pain management outcomes varied; VR was effective during procedures like IV cannulation in some studies but showed no significant differences in others.

**Table 3:** Characteristics of included studies RCT: Randomized Controlled Trial, VR: Virtual Reality, EG: Experimental Group, CG: Control Group, m-YPAS: Modified Yale Preoperative Anxiety Scale, ED: Emergence Delirium, BAI: Beck Anxiety Inventory, EMLA: Eutectic Mixture of Local Anesthetics, GA: General Anesthesia

First Author, Year	Intervention of EG1	Intervention of EG2	Intervention of CG	Period of Intervention	Outcome measures	Results
Jung-Hee Ryu, 2017 [5]	Immersive VR tour of the operating theater.	-	Conventional education regarding the perioperative process.	1 hour prior to surgery	• Incidence and severity of emergence delirium (ED) • Preoperative anxiety using m-YPAS • Postoperative behavioral disturbances.	No significant reduction in the incidence and severity of ED between groups. Significant reduction in preoperative anxiety in the VR group compared to the control group (mean difference of 9.2, P = 0.022). No significant difference in postoperative behavioral disturbances between groups at 1 day and 14 days.
Fateme Dehghan, 2019 [14]	Virtual reality (VR) environment simulating the steps of going to the operating room using VR goggles and headphones. They were immersed in a virtual environment that involved visual and auditory senses.	-	Did not receive any VR exposure; instead, parents were requested to touch and caress their children before surgery.	The VR exposure lasted for 5 minutes prior to the surgery.	• Preoperative anxiety using m-YPAS	EG showed significant reductions in anxiety scores in all domains except for the arousal domain (p < 0.05). CG also showed reduction in anxiety. In comparison, anxiety scores in the control group did not show significant reductions. The VR intervention was effective in reducing preoperative anxiety in children.
Jung-Hee Ryu, 2022 [13]	VR tour of the operating room (OR) a few days before anesthesia,	VR tour of the OR 10 minutes before anesthesia.	Standard verbal information about the anesthesia and surgery process 10 minutes before anesthesia.	A few days before anesthesia for the VR A group, 10 minutes before anesthesia for the VR B group	• Preoperative anxiety of children (m-YPAS) • Anxiety of caregivers (BAI) • Caregivers’ satisfaction with the overall process of anesthesia and surgery	VR B group had significantly lower preoperative anxiety (m-YPAS) than the control group and VR A group (p = 0.001). There was no significant difference in caregivers’ anxiety (BAI) across the groups (p = 0.605). Caregivers’ satisfaction was higher in the VR A group compared to the control (p = 0.014).
Rishabh Ravi, 2023 [16]	VR headset powered by a smartphone playing videos (such as swimming with dolphins or playing cricket). The VR was used during intravenous (IV) cannulation and separation from parents before surgery.	-	EMLA cream (a topical anesthetic) was applied 1 hour prior to IV cannulation at the site of cannulation.	During intravenous cannulation and preoperative parent separation.	• Pain during IV cannulation (measured with Faces Pain Scale). • Parental separation anxiety (measured with a parental separation anxiety scale). • Heart rate and oxygen saturation were monitored 10 minutes pre-procedure, during the procedure, and 10 minutes post-procedure.	The VR group had a lower Faces Pain Scale score as compared to the EMLA patch group (5.31 ± 1.31 vs. 6.54 ± 1.16). The VR group had lower parental separation anxiety scores compared to the EMLA group (2.51 ± 0.97 vs. 3.53 ± 0.50). The VR group exhibited more stable heart rates and oxygen saturation levels during the procedure as compared to the EMLA group.
Michael J. Jung, 2021 [15]	VR headset that provided immersive audiovisual distraction during induction of general anesthesia (GA). The VR headset displayed a pre-selected interactive game designed for pediatric perioperative use.	-	Patients received standard medical care without any audiovisual devices during induction of GA.	During induction of general anesthesia in the operating room.	• Parental anxiety (State-Trait Anxiety Inventory), pediatric induction compliance, and parental satisfaction. • Preoperative anxiety of children (m-YPAS)	The VR group had significantly lower mYPAS scores during induction compared to the control group (0.0 (0.0–5.0) vs. 13.3 (5.0–26.7), p < 0.0001). In the mixed-effects model, the VR group had an estimated 14.5-point lower mYPAS score (95% CI: 9.3–19.8, p < 0.0001) at induction. No significant differences in parental anxiety, pediatric induction compliance, or parental satisfaction between the groups.

Quantitative synthesis revealed a pooled standardized mean difference (SMD) of -0.73, favoring VR interventions for reducing preoperative anxiety. This effect was statistically significant, with moderate heterogeneity (I² = 67%) among studies. The variability in effect sizes did not undermine the overall conclusion of VR’s moderate effectiveness in anxiety reduction, as the pooled analysis consistently demonstrated favorable outcomes for the VR groups.

Discussion

This systematic review and meta-analysis examined the effectiveness of VR in reducing preoperative anxiety in pediatric surgical patients. Preoperative anxiety is a significant issue, affecting children's psychological well-being, surgical experience, and recovery. Traditional pharmacological approaches, such as midazolam, have side effects like respiratory depression and delayed recovery, making non-pharmacological methods preferable. VR has emerged as a promising, non-invasive tool, providing immersive, interactive environments to distract children and reduce anxiety without the risks of sedative medications.

The review included five RCTs that evaluated VR interventions across different surgical contexts. Three studies were included in the meta-analysis, which found a moderate and significant reduction in preoperative anxiety with VR compared to traditional care or other non-pharmacological interventions (SMD = -0.73). Most studies were of good to excellent quality, though limitations in external validity due to small sample sizes and specific hospital settings were noted.

While the studies consistently reported reductions in anxiety, there was variability in outcomes like postoperative pain and emergence delirium. VR interventions with interactive content, such as games, appeared more effective than simpler VR environments, highlighting the importance of VR content in managing anxiety. Other research has suggested that VR can also improve postoperative recovery, but this review focused on its preoperative effects.

Several limitations were noted in this review. Small sample sizes (40-105 participants) reduced the statistical power, and the moderate heterogeneity (I² = 67%) across studies suggests variability in the effectiveness of VR interventions. Additionally, long-term effects on postoperative outcomes and patient satisfaction remain unclear, as most studies did not report follow-up data. Furthermore, adverse events, such as motion sickness, were rarely mentioned.

## Conclusions

In conclusion, this systematic review and meta-analysis demonstrates that VR is an effective tool for reducing preoperative anxiety in pediatric patients. The immersive and engaging nature of VR allows children to focus on a virtual environment, diverting their attention from the stress of the surgical setting. The studies included in this review consistently reported reductions in anxiety, though the degree of effectiveness varied depending on the type of VR intervention and the surgical context. While VR holds great promise as a non-pharmacological intervention, further research is needed to standardize its use, explore its long-term benefits, and assess its broader applicability in pediatric healthcare. If successfully implemented, VR could revolutionize the way pediatric anxiety is managed in perioperative care, offering a safe and effective alternative to traditional methods.
